# Factors that influence in-brace derotation effects in patients with adolescent idiopathic scoliosis: a study based on EOS imaging system

**DOI:** 10.1186/s13018-024-04789-7

**Published:** 2024-05-12

**Authors:** Qing Fan, Jingfan Yang, Lin Sha, Junlin Yang

**Affiliations:** Xinhua Hospital affiliated to Shanghai Jiao tong University School of Medicine, Shanghai, China

**Keywords:** Adolescent idiopathic scoliosis, Derotation effects, Brace treatment, EOS imaging system

## Abstract

**Objective:**

To investigate the effects of bracing on apical vertebral derotation and explore the factors that influence in-brace derotation effects in adolescent idiopathic scoliosis (AIS) patients.

**Summary of background data:**

For patients with AIS, vertebral rotation causes cosmetic appearance abnormalities and acts as an indicator for curve progression. However, there have been few studies investigating the precise derotation effects of bracing for apical vertebra. The application of EOS imaging system enables quantitative evaluation of vertebral rotation in the axial plane in a standing position.

**Methods:**

There were 82 eligible patients enrolled in current study, who underwent EOS imaging evaluation before and immediately after bracing. The clinical demographic data (age, gender, Risser sign and menstrual status) were recorded. The correlation analyses between derotation effects and key parameters (age, pre-brace Cobb angle, thoracic kyphosis, lumbar lordosis, vertebral rotation, pelvis axial rotation and apical vertebral level) were performed. The in-brace derotation effects stratified by gender, Risser sign, apical vertebral level, menarche status, coronal balance and sagittal balance were also analyzed.

**Results:**

The rotation of apical vertebra was decreased from 8.8 ± 6.0 degrees before bracing to 3.8 ± 3.3 degrees immediately after bracing (*p* < 0.001), and the derotation rate was 49.2 ± 38.3%. The derotation degrees in brace was significantly correlated with major curve Cobb angle (r = 0.240, *p* = 0.030), minor curve Cobb angle (r = 0.256, *p* = 0.020) and total curve Cobb angle (r = 0.266, *p* = 0.016). Both the pre-brace apical vertebral rotation and apical vertebral level were significantly correlated with derotation effects in brace (*p* < 0.001). Patients with thoracic major curve showed worse derotation effects than those with lumbar major curve (*p* < 0.001). In addition, patients with coronal balance showed better in-brace derotation effects than those with coronal decompensation (*p* = 0.005).

**Conclusions:**

A satisfactory apical vertebral derotation rate (approximately 50%) could be obtained immediately after bracing in AIS patients. Pre-brace Cobb angle of curve, pre-brace apical vertebral rotation, apical vertebral level and coronal balance exhibited close associations with in-brace derotation effects of apical vertebra.

## Introduction

Adolescent idiopathic scoliosis (AIS) is a complex three-dimensional spinal deformity which occurs in adolescence with unknown etiology [1]. The overall incidence of AIS can reach 2–3% in adolescents, while the incidence in females is 1.5–3 times higher than that in males [2, 3]. About two-thirds of patients will experience continued progression of spinal deformity during rapid growth period, which not only affects appearance perception and causes psychological disorders, but may also leads to spinal degeneration and even cardiopulmonary dysfunction [3]. Therefore, a standard treatment strategy should be performed to protect mental and physical health of patients with AIS.

With its satisfactory therapeutic effects, bracing stands as one of the commonly used interventions for conservative treatments of AIS [4]. Numerous studies have demonstrated that bracing can arrest the progression of spinal curvature and correct coronal curve by external force [4, 5]. However, very limited studies focus on vertebral derotation effects of bracing [6, 7]. Vertebral rotation leads to cosmetic appearance abnormalities including razorback and uneven waist, which inevitably impairs patients’ appearance perception. Furthermore, insufficient derotation effects also increase failure rate of brace treatment [8, 9]. Thus, more attention should be paid on the effects of bracing on vertebral derotation.

The EOS imaging system has been widely used to evaluate spinal morphology recently [10, 11]. It allows three-dimensional modeling of spine in a standing position with a low-does X-ray system. The application of EOS imaging system enables quantitative evaluation of vertebral rotation in the axial plane in a standing position [11–13]. Thus, the current study was performed to investigate the effects of bracing on apical vertebral derotation and explore the factors that influence in-brace derotation effects in AIS. The hypotheses of this study were that brace treatment could contribute to apical vertebral derotation, and there existed several meaningful parameters to predict the derotaion effects of bracing.

## Materials and methods

### Subjects

This study has been approved by local ethics committee (Approval No. XHEC-D-2021-150) and was strictly complied with the Declaration of Helsinki. The current retrospective study enrolled patients with AIS who underwent brace treatment from September 2019 to June 2021 in our institution. The detailed inclusion criteria were as follows: (1) diagnosed with AIS with age > 10 years old; (2) treated with modified Gensingen braces [14]; (3) Risser sign no more than stage 3 [7, 14]; (4) underwent EOS imaging evaluation in a standing position before and immediately after bracing. Finally, eighty-two eligible patients (68 females and 14 males, mean age 13.5 ± 1.5 years old) were included in current study.

### Clinical and radiological assessment

**Fig. 1:**
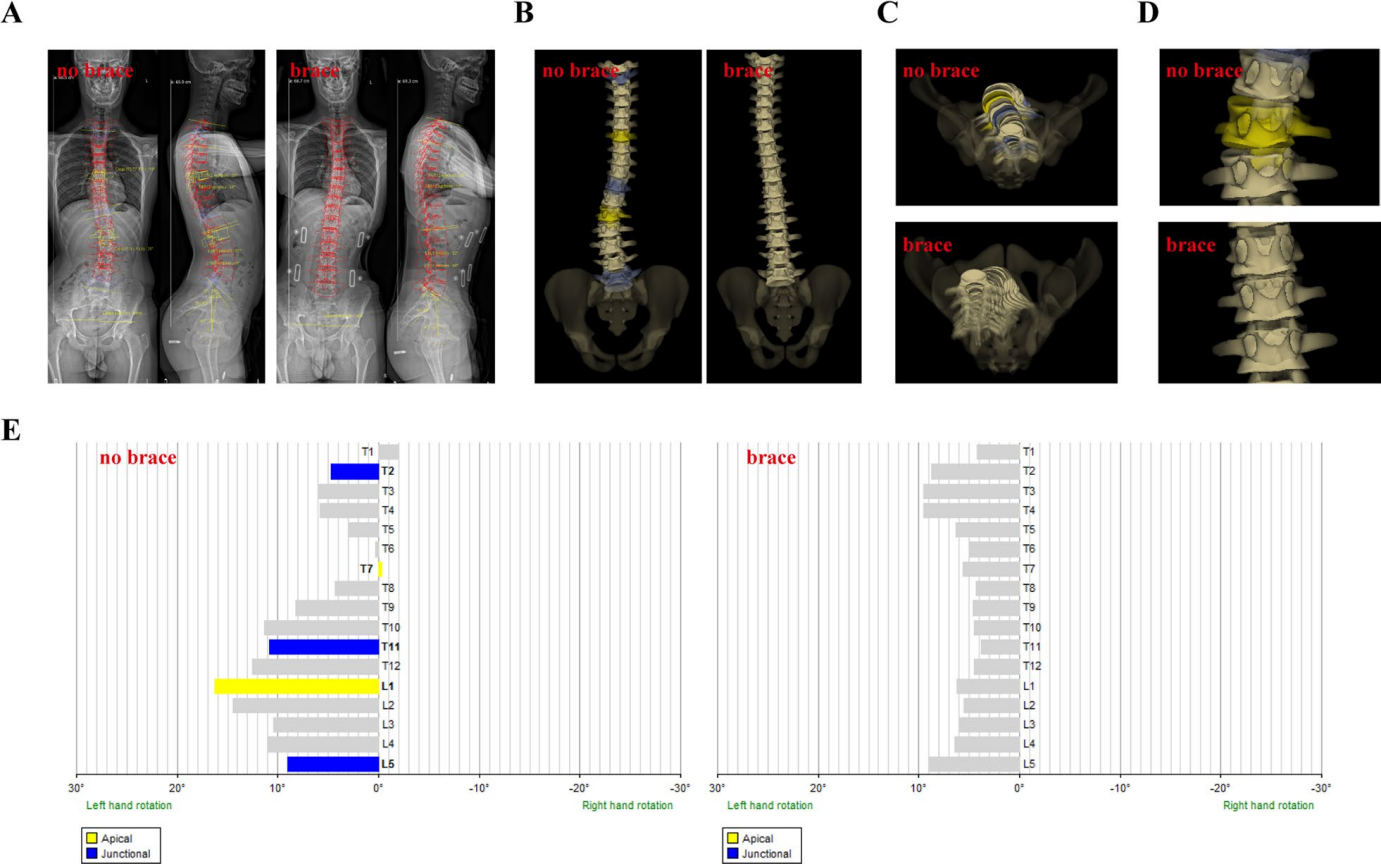
An illustrative case of EOS 3D reconstruction of an AIS patient before and immediately after bracing. **A** Anteroposterior view; **B** 3D images from the front; **C** 3D images from the above; **D** Enlarged 3D image view of apical vertebra; **E** Diagrams illustrating vertebral rotation

The clinical demographic data (age, gender, Risser sign and menstrual status) were recorded at the time of EOS imaging. EOS imaging system was used to acquire biplanar anteroposterior and lateral images of full spine in a standing position. An initial EOS image of full spine was obtained for patients without a brace. Then the brace was immediately applied, and the second EOS images for patients wearing brace was obtained (Fig. 1A). Subsequently, the 3D model of the whole spine was constructed by matching the anatomic landmarks on EOS imaging working position (Fig. 1B–D). After three-dimensional reconstruction by EOS imaging system, the coronal Cobb angles, thoracic kyphosis (T4–T12), lumbar lordosis (L1–L5), pelvis axial rotation and vertebral rotation in the axial plane (Fig. 1E) could be automatically obtained. The coronal imbalance was defined as the distance between the plumb line from the center of C7 and midline of sacrum more than 2 cm. The sagittal imbalance was defined as the distance between the posterior superior corner of S1 to the plum line drawn from center of C7 more than 4 cm [15]. All the parameters before and immediately after bracing were documented. Derotation degrees and derotation rate in brace were calculated using following formula: In-brace derotaion degrees =|rotation degrees before bracing| − |rotation degrees immediately after bracing|; In-brace derotation rate = (in-brace derotation degrees)/|pre-brace rotation degrees| * 100%.

### Statistical analysis

Statistical analysis was conducted using SPSS version 20.0 for Windows (SPSS Inc., Chicago, IL, USA). The Shapiro–Wilk test was used to determine normality of continuous data, which were presented as mean ± standard deviation. The paired Student t test was selected to compare parameters before bracing and immediately wearing bracing. The Pearson correlation analysis between derotation effects and key parameters (age, pre-brace major curve Cobb angle, minor curve Cobb angle, total curve Cobb angle, thoracic kyphosis, lumbar lordosis, vertebral rotation, pelvis axial rotation and apical vertebral level) were performed. Independent t test or Wilcoxon rank-sum test was performed to compare groups stratified by different factors. It was considered as significance if *p* value was less than 0.05.

## Results

### Therapeutic effects of bracing

The therapeutic effects of bracing were presented in Table 1. The major curve Cobb angle was decreased from 32.7 ± 9.7 degrees before bracing to 14.5 ± 11.7 degrees immediately after bracing (*p* < 0.001), with in-brace correction rate of 56.9 ± 32.9% (Table 1). The minor curve Cobb angle was reduced from 22.5 ± 10.3 degrees before bracing to 11.7 ± 9.5 degrees immediately after bracing (*p* < 0.001), with in-brace correction rate of 46.6 ± 53.8% (Table 1). The total curve Cobb angle was improved from 55.2 ± 18.6 degrees before bracing to 26.2 ± 18.5 degrees immediately after bracing (*p* < 0.001), and the in-brace correction rate was 52.8 ± 31.1% (Table 1). Thoracic kyphosis was decreased from 19.1 ± 8.6 degrees before bracing to 15.5 ± 9.8 degrees immediately after bracing (*p* < 0.001), with in-brace correction loss of 21.2 ± 29.3% (Table 1). The pre-brace lumbar lordosis was 46.5 ± 10.5 degrees and in-brace lumbar lordosis was 34.8 ± 11.5 degrees, with in-brace correction loss of 25.8 ± 18.4% (Table 1). As for derotation effects, the rotation of apical vertebra was decreased from 8.8 ± 6.0 degrees before bracing to 3.8 ± 3.3 degrees immediately after bracing (*p* < 0.001), and the derotation rate was 49.2 ± 38.3% (Table 1).

**Table 1:** Pre- to in-brace changes of three-dimensional parameters

Parameters	Before brace	In brace	△Correction	Correction rate/correction loss	*p* value
Major curve Cobb angle	32.7 ± 9.7	14.5 ± 11.7	18.3 ± 9.9	56.9 ± 32.9	< 0.001*
Minor curve Cobb angle	22.5 ± 10.3	11.7 ± 9.5	12.3 ± 8.7	46.6 ± 53.8	< 0.001*
Total curve Cobb angle	55.2 ± 18.6	26.2 ± 18.5	29.2 ± 17.1	52.8 ± 31.1	< 0.001*
Rotation of apical vertebra	8.8 ± 6.0	3.8 ± 3.3	5.2 ± 4.3	49.2 ± 38.3	< 0.001*
Thoracic kyphosis	19.1 ± 8.6	15.5 ± 9.8	3.5 ± 5.3	21.2 ± 29.3	< 0.001*
Lumbar lordosis	46.5 ± 10.5	34.8 ± 11.5	11.7 ± 7.8	25.8 ± 18.4	< 0.001*

Values are presented as mean ± SD. *Statistically significant

### Correlation analyses between derotation effects and key parameters

**Fig. 2:**
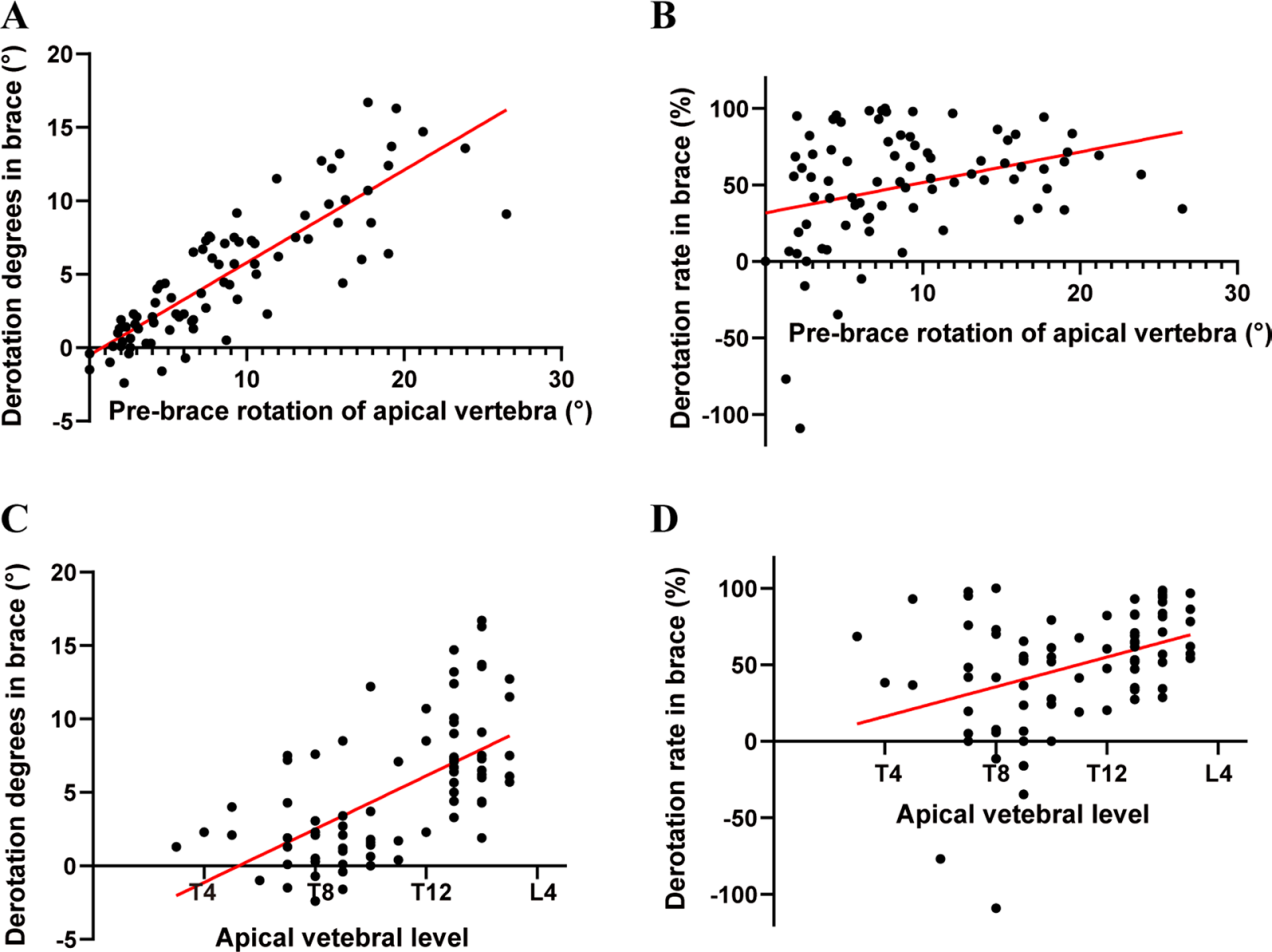
Scatterplots and regression line between derotation effects and related parameters. **A** Scatterplots and regression line between pre-brace rotation of apical vertebra and derotation degrees in brace (r = 0.846, *p* < 0.001); **B** Scatterplots and regression line between pre-brace rotation of apical vertebra and derotation rate in brace (r = 0.315, *p* = 0.004); **C** Scatterplots and regression line between apical vertebral level and derotation degrees in brace (r = 0.610, *p* < 0.001); **D** Scatterplots and regression line between apical vertebral level and derotation rate in brace (r = 0.384, *p* < 0.001)

The correlation analyses between derotation effects and key parameters were presented in Table 2. There were no correlations between derotation effects and age, thoracic kyphosis, lumbar lordosis, pelvis axial rotation (Table 2). The derotation degrees in brace was significantly correlated with major curve Cobb angle (r = 0.240, *p* = 0.030), minor curve Cobb angle (r = 0.256, *p* = 0.020) and total curve Cobb angle (r = 0.266, *p* = 0.016) (Table 2). However, there were no correlations between derotation rate in brace and major curve Cobb angle (r = 0.034, *p* = 0.758), minor curve Cobb angle (r = 0.084, *p* = 0.455) and total curve Cobb angle (r = 0.064, *p* = 0.567) (Table 2). Furthermore, the pre-brace rotation of apical vertebra was significantly correlated with derotation degrees in brace (r = 0.846, *p* < 0.001) and derotation rate in brace (r = 0.315, *p* < 0.001) (Table 2 and Fig. 2A, B). There was also close correlation between apical vertebral level and derotation degrees in brace (r = 0.610, *p* < 0.001), as well as apical vertebral level and derotation rate in brace (r = 0.384, *p* < 0.001) (Table 2 and Fig. 2C, D).

**Table 2:** Correlation analyses between derotation effects and key parameters

Parameters	Derotation degrees in brace		Derotation rate in brace	
	r	*p* value	r	*p* value
Age	0.102	0.362	− 0.021	0.854
Major curve Cobb angle	0.240	0.030*	0.034	0.758
Minor curve Cobb angle	0.256	0.020*	0.084	0.455
Total curve Cobb angle	0.266	0.016*	0.064	0.567
Thoracic kyphosis	− 0.040	0.718	0.028	0.802
Lumbar lordosis	− 0.060	0.595	0.156	0.163
Rotation before brace	0.846	< 0.001*	0.315	< 0.001*
Pelvis axial rotation	0.164	0.141	0.116	0.301
Apical vertebral level	0.610	< 0.001*	0.384	< 0.001*

Values are presented as mean ± SD. *Statistically significant

### In-brace derotation effects stratified by different factors

The in-brace derotation effects stratified by different factors were presented in Table 3. There were no significant differences of derotation effects in different genders, different stages of Risser sign, different menarche statuses, and different statuses of sagittal balance (Table 3). However, patients with thoracic major curve showed worse derotation effects than those with lumbar major curve (2.6 ± 3.3° vs. 8.3 ± 3.8° of derotation degrees in brace, *p* < 0.001; 36.0 ± 42.8% vs. 67.0 ± 22.2% of derotation rate in brace, *p* < 0.001) (Table 3). In addition, patients with coronal balance also showed significantly better in-brace derotation rate than those with coronal decompensation (55.1 ± 35.4% vs. 25.2 ± 42.4%, *p* = 0.005) (Table 3).

**Table 3:** In-brace derotation effects stratified by different factors

Parameters		Patients	Derotation degrees in brace (°)		Derotation rate in brace (%)	
Gender	Male	14	4.1 ± 3.3	*p* = 0.422	48.3 ± 34.5	*p* = 0.580
	Female	68	5.0 ± 4.5		49.2 ± 38.3	
Risser sign	0.1	36	5.0 ± 4.8	*p* = 0.951	53.3 ± 45.6	*p* = 0.407
	2.3	46	5.1 ± 4.3		46.1 ± 32.0	
Apical vertebral level	Thoracic	47	2.6 ± 3.3	*p* < 0.001*	36.0 ± 42.8	*p* < 0.001*
	Lumbar	35	8.3 ± 3.8		67.0 ± 22.2	
Menarche status	Premenarcheal	14	4.4 ± 3.4	*p* = 0.489	60.3 ± 33.9	*p* = 0.280
	Postmenarcheal	54	5.4 ± 5.0		47.7 ± 39.4	
Coronal balance	Balance	66	5.3 ± 4.4	*p* = 0.301	55.1 ± 35.4	*p* = 0.005*
	Imbalance	16	4.0 ± 5.2		25.2 ± 42.4	
Sagittal balance	Balance	67	5.0 ± 4.6	*p* = 0.886	50.3 ± 40.7	*p* = 0.610
	Imbalance	15	5.2 ± 4.2		44.6 ± 27.4	

Values are presented as mean ± SD. *Statistically significant

## Discussion

The current study investigated the effects of bracing on apical vertebral derotation by applying EOS imaging system. Factors which influenced in-brace derotation effects were also explored. A satisfactory apical vertebral derotation rate (approximately 50%) could be obtained immediately after bracing. It was identified that pre-brace Cobb angle of curve, pre-brace apical vertebral rotation, apical vertebral level and coronal balance were significantly correlated with in-brace derotation effects of apical vertebra.

AIS is a complex three-dimensional spinal deformity characterized by a Cobb angle greater than 10° [15]. Vertebral rotation causes cosmetic appearance abnormalities including thoracic cage asymmetry and uneven waist. In addition, it serves as an indicator for curve progression, and insufficient derotation effect also contributes to failure of brace treatment [8, 9]. Thus, it is of great importance to investigate the effects of bracing on axial plane, as well as the factors that influence in-brace derotation effects. However, correction of vertebral rotation in the axial plane is not always the primary focus of brace treatment. To the best of our knowledge, only Baymurat et al. investigated the effects of bracing on vertebral rotation [6]. Using Nash and Moe classification, they found that Boston brace could significantly reduce apical vertebral rotation in AIS patients [6]. However, the evaluation of vertebral rotation by Nash and Moe was not a quantitative measurement method.

Computed tomography (CT) images were typically used to quantitatively analyze the vertebral rotation [11]. However, obtaining images in the standing position is not feasible with CT since it is performed in the supine position, leading to a significant underestimation of vertebral rotation. In addition, the determination of vertebral rotation angle using the reference of the radiographic sagittal plane ignores the pelvic rotational position [16]. The high radiation exposure also impeded the wide application of CT for vertebral rotation evaluation. Thus, other techniques are required to precisely evaluate the vertebral rotation in the axial plane in a standing position. The introduction of EOS imaging system to clinic makes quantitative measurement of vertebral rotation more feasible [11–13]. EOS is a relative new technique which can simultaneously acquire biplanar anteroposterior and lateral images in a standing position, with radiation exposure only one-tenth of the conventional radiography [17]. Moreover, the high measurement reliability of rotational deformity by EOS has been demonstrated by several investigations [11], which is comparable to CT measurement [18]. Thus, the application of EOS imaging system enhances the credibility of the conclusions drawn from the current study.

The current study found that approximately 50% vertebral derotation rate could be achieved immediately after bracing. Mechanistically, brace corrects spinal deformities by distracting concave side and compressing convex side, thereby reinstating the normal alignment of the spine in coronal planes [19]. The effectiveness of brace in correcting coronal deformity of AIS has been well demonstrated [4, 6]. As for sagittal plane, there was significant correction loss of thoracic kyphosis and lumbar lordosis after bracing in current study, which was similar with previous finding that brace treatment could flatten these two parameters [20, 21]. Although progressive modifications of spine may occur during treatment period [21], long-term effects of bracing on sagittal alignment for patients in current study should be further investigated. In addition, only Baymurat et al. investigated detailed derotation effects of brace treatment and they found that the apical vertebral rotation improved from 2.1 ± 0.6 before bracing to 1.1 ± 0.5 after bracing by using Nash and Moe classification evaluation [6]. To the best of our knowledge, the current study is the first investigation to illustrate the detailed quantitative derotation rate of apical vertebra.

The current study also demonstrated that pre-brace Cobb angle of curve, pre-brace apical vertebral rotation, apical vertebral level and coronal balance were significantly correlated with in-brace derotation effects of apical vertebra. Since Cobb angle of curve and apical vertebral rotation were closely related [22], it was reasonable to find that both pre-brace Cobb angle and apical vertebral rotation were positively correlated with derotation effects. However, it is worth noting that in-brace derotation effects were not significantly correlated with thoracic kyphosis or lumbar lordosis. Since the patients in currents study showed no abnormal sagittal alignment, we supposed that there was limited association between vertebral rotation and sagittal profile. In addition, apical vertebra of major curve located in thoracic area showed worse derotation effects in brace, which could be resulted from resistance of rib cage and related tissues attached in thoracic vertebra. This finding indicated that more treatment strategies should be focused on correcting thoracic apical vertebra other than brace treatment. Furthermore, patients with coronal balance shower better in-brace derotation effects. Given that AIS is a complex three-dimensional deformity of spine, decompensated scoliosis may also influence axial plane and increase difficulty of vertebra derotation.

The present study was not without limitations. Firstly, this was a retrospective study which would be affected by some inherent biases. Secondly, we only analyzed the data before bracing and immediately after bracing. Since the in-brace torsional correction may be lost with time, the conclusion in current study should be applied exclusively to this time interval. Thirdly, EOS system is not routinely used in clinical setting in the majority of hospitals, which may limit the generalizability of current study’s finding in daily practice decision-making. In addition, the current study failed to include curve flexibility when considering additional radiation exposure. Curve flexibility is closely associated with Cobb angle correction, so it may also be a crucial factor in determining in-brace vertebral derotation effects. Thus, further study should be performed to include information of curve flexibility and investigate the results of long-term follow-up of in-brace derotation effects.

## Conclusions

In conclusion, a satisfactory apical vertebral derotation rate (approximately 50%) could be obtained immediately after bracing in AIS patients. Pre-brace Cobb angle of curve, pre-brace apical vertebral rotation, apical vertebral level and coronal balance exhibited close associations with in-brace derotation effects of apical vertebra.
