# Correction: Factors that influence in-brace derotation effects in patients with adolescent idiopathic scoliosis: a study based on EOS imaging system

**DOI:** 10.1186/s13018-025-06645-8

**Published:** 2026-03-13

**Authors:** Qing Fan, Jingfan Yang, Lin Sha, Junlin Yang

**Affiliations:** Xinhua Hospital Affiliated to Shanghai Jiao Tong University Schoolof Medicine, Shanghai, China

**Correction: Journal of Orthopaedic Surgery and Research (2024) 19:293** 10.1186/s13018-024-04789-7.

In this article [1], the authors regret the incorrect use of the term “modified Gensingen Brace” in the original publication. The correct terminology should be “Chêneau-type braces.” The authors apologize for this error.
